# Genomes of enteric pathogen isolates from polymicrobial infections in cases of pediatric diarrhea

**DOI:** 10.1128/mra.01015-25

**Published:** 2025-12-05

**Authors:** Bernadette A. Hritzo, Jane M. Michalski Wilhelm, David A. Rasko

**Affiliations:** 1 Institute for Genome Sciences, University of Maryland School of Medicine 12264, Baltimore, Maryland, USA; 2 Department of Microbiology and Immunology, University of Maryland Baltimore School of Medicine 12264, Baltimore, Maryland, USA; 3 Department of Microbial Pathogenesis, University of Maryland School of Dentistry 89015, Baltimore, Maryland, USA; 4 Center for Pathogen Research, University of Maryland School of Medicine 12264, Baltimore, Maryland, USA; University of Pittsburgh School of Medicine, Pittsburgh, Pennsylvania, USA

**Keywords:** polymicrobial, enteric, genome

## Abstract

The majority of human enteric infections are not caused by a single pathogen. We describe the genomes of *Escherichia coli, Shigella* spp.*,* and *Aeromonas* spp. isolates from polymicrobial infections in four subjects enrolled in the Global Enteric Multicenter Study.

## ANNOUNCEMENT

Polymicrobial enteric pathogen infections are often observed in surveillance studies (1–3). In this announcement, we describe the genomes of *Escherichia coli, Shigella* spp., and *Aeromonas* spp. from polymicrobial infections of four subjects in the Global Enteric Multicenter Study.

Initial isolation was conducted as described by Panchalingam *et al.* (4). Briefly, fecal samples transported in cold containers were inoculated into Cary-Blair transport media within 6 and 18 h of collection, sub-cultured onto selective growth media, and incubated 48 hours at 35°C–37°C described below.

Dark green colonies, with darker green centers, were selected from Ryan agar and sub-cultured on trypticase soy agar (TSA). Single colonies were examined for 2, 4-diamino-6, 7-diisopropyl pteridine (O/129) resistance (5), salt tolerance (cultured with 0%, 6%, and 8% NaCl in TSA at 37°C for 24 h), and oxidase and catalase activities. O/129-resistant, oxidase(+), and catalase(+) isolates, which grew in 0% NaCl only, were designated *Aeromonas* spp.

Several pink/red lactose-fermenting colonies were selected from McConkey agar and sub-cultured in motility indole ornithine (MIO) medium. Indole(−) cultures were assessed by Methyl Red, Voges Proskauer, and Citrate tests. Suspected *E. coli* isolates (methyl-red(+), Voges Proskauer(−), citrate(−)) were categorized into pathogenic types by multiplex PCR (6).

Single colorless, non-lactose-fermenting colonies were selected from McConkey agar and sub-cultured in triple-sugar iron (TSI), MIO, lysine decarboxylase (LD), citrate, and urea biochemical typing media. Isolates identified as *Shigella* spp. (TSI(+), MIO(−), LD(+), Citrate(−), Urea(−)) were serotyped with polyvalent group A, B, C, and D (Denka Seiken/Reagensia).

Verified isolates of each species were frozen in 20% glycerol and maintained at −80°C. Frozen cultures were provided to the Center for Vaccine Development at the University of Maryland School of Medicine.

Isolates were resurrected from frozen culture on L-agar overnight at 37°C. Genomic DNA was extracted using Qiagen’s DNeasy Blood & Tissue kit (Cat#69504), and paired-end sequencing libraries were generated with unsheared genomic DNA. Nanopore libraries were prepared using Oxford Nanopore’s “Genomic DNA by Ligation” kit (Cat# SQK-LSK110) and protocol (7) and run on Nanopore R9 flow cells; basecalling was performed using Guppy (v4.2.2) (8). Illumina libraries were prepared using the Kapa library kit (Roche/Illumina) and sequenced with paired-end 150 bp reads on the Illumina HiSeq 4000 platform. Quality control and adapter trimming were performed with bcl2fastq (v2.20.0.445) (9) and porechop (v0.2.3_seqan2.1.1) (10) for Illumina and ONT sequencing, respectively. Hybrid assembly using both Illumina and ONT reads was conducted with Unicycler (v0.4.8) (11). The final assembly statistics were recorded with QUAST (5.0.2) (12). Software was run with default values and kits as per the manufacturer’s instructions. Table 1 contains the total number of reads generated for each isolate, sequencing technology, relevant sequencing statistics, and assembly GenBank accession numbers.

**TABLE 1:** Isolate and sequencing features^*b*^

Isolate features					Sequencing results													Accession Numbers			
Sample	Country	Sex	Isolate	Species (Pathotype/Serotype)	Molecule ID	Molecule type	Size (bp)	Assembly	Illumina read pairs	Illumina bases	ONT trimmed reads	ONT trimmed bases	ONT N50	coverage	# molecules	Total genome length (bp)	GC (%)	BioProject	Assembly	SRA (Illumina, ONT)	
600239	Bangladesh	Female	A600239	*Aeromonas* *caviae*	A600239	Chromosome	4,678,049	A600239	2000385	604116270	865542	2296477342	2636.6	620	1	4678049	61.25	PRJNA1252136	CP189711	SRR33210438	SRR33210434
			E600239	*E. coli*	E600239	Chromosome	5,193,357	E600239	1641473	495724846	2174494	4966171119	2284.3	1013.6	4	5388457	50.71	PRJNA1252136	CP189704	SRR33210420	SRR33210430
				enteroaggregative *E. coli* ^*a*^	pE600239_118	Plasmid - *bfp*-like cluster	118,072												CP189705		
					pE600239_74	Plasmid - conjugation	74,407												CP189706		
					pE600239_3	Cryptic plasmid	2,621												CP189707		
			S600239	*Shigella flexneri* PG1^*a*^	S600239	Chromosome	4,583,497	S600239	1874954	566236108	960086	4500110194	4680.2	1052.7	6	4812595	50.63	PRJNA1252136	CP189688	SRR33210416,	SRR33210425
					pS600239_217	Virulence plasmid	217,583												CP189689		
					pS600239_4	Cryptic plasmid	4,110												CP189690		
					pS600239_3	Cryptic plasmid	3,177												CP189691		
					pS600239_2	Cryptic plasmid	2,690												CP189692		
					pS600239_1	cryptic plasmid	1,538												CP189693		
600717	Bangladesh	Male	A600717	*Aeromonas veronii*	A600717	Chromosome	4,707,010	A600717	1886182	569626964	264889	1740021909	6556.1	490.7	1	4707010	58.59	PRJNA1252136	CP189710	SRR33210437	SRR33210433
			E600717	*E. coli*	E600717	Chromosome	5,020,477	E600717	1808644	546210488	714650	2375845449	3326	581.6	2	5024039	50.66	PRJNA1252136	CP189702	SRR33210419	SRR33210429
				Atypical enteropathogenic *E. coli^a^*	pE600717_4	Cryptic plasmid	3,562												CP189703		
			S600717	*Shigella flexneri* serotype 6	600717	Chromosome	46,51,652	S600717	1734941	523952182	601973	2145988398	3553.1	550.7	5	4848082	51	PRJNA1133736	CP162048	SRR29762656	SRR29762665
					p600717_186	Virulence plasmid	1,86,903												CP162049		
					p600717_4	Cryptic plasmid	4,065												CP162050		
					p600717_2 a	Cryptic plasmid	2,783												CP162051		
					p600717_2b	Cryptic plasmid	2,679												CP162052		
603020	Bangladesh	Female	A603020	*Aeromonas veronii*	A603020	Chromosome	4,498,453	A603020	4354804	1315150808	239700	1360614937	5668.9	594.1	2	4503839	58.85	PRJNA1252136	CP189709	SRR33210426	SRR33210432
			E603020	*E. coli*	E603020	Chromosome	4,905,389	E603020	1558815	470762130	424088	2834875313	6681.8	658.2	2	5022037	50.63	PRJNA1252136	CP189700	SRR33210436	SRR33210428
				Enterotoxigenic *E. coli*^*a*^	pE603020_117	Plasmid - encodes LT	116,648												CP189701		
			S603020	*Shigella flexneri* serotype 6	603020	Chromosome	46,83,211	S603020	2114734	638649668	915878	3331874243	3640.8	813.2	6	4882607	50.98	PRJNA1133736	CP162042	SRR29762655	SRR29762664
					p603020_184	Virulence plasmid	1,84,337												CP162043		
					p603020_7	Cryptic plasmid	6,768												CP162044		
					p603020_4	Cryptic plasmid	4,066												CP162045		
					p603020_2	Cryptic plasmid	2,679												CP162046		
					p603020_1	Cryptic plasmid	1,546												CP162047		
700241	Pakistan	Male	A700241	*Aeromonas caviae*	A700241	Chromosome	4,448,984	A700241	3910809	1181064318	770522	3280504464	4249.1	1001.6	2	4454370	61.88	PRJNA1252136	CP189708	SRR33210421	SRR33210431
			E700241	*E. coli*	E700241	Chromosome	4,933,650	E700241	1875846	566505492	488257	3056731052	6262.2	697	6	5198588	50.58	PRJNA1252136	CP189694	SRR33210417	SRR33210427
				Enterotoxigenic *E. coli*^*a*^	pE700241_161	Plasmid - encodes LT	161,662												CP189695		
					pE700241_57	Plasmid - EatA	57,131												CP189696		
					pE700241_28	Plasmid	28,461												CP189697		
					pE700241_7	Cryptic plasmid	6,646												CP189698		
					pE700241_6	Cryptic plasmid	5,538												CP189699		
			S700241	*Shigella flexneri* PG3^*a*^	S700241	Chromosome	4,659,789	S700241	1905009	575312718	248877	1197245105	4800.1	362.1	5	4895304	50.63	PRJNA1252136	CP189683	SRR33210435	SRR33210422
					pS700241_222	Virulence plasmid	222,091												CP189684		
					pS700241_6	Cryptic plasmid	6,200												CP189685		
					pS700241_4	Cryptic plasmid	4,042												CP189686		
					pS700241_3	Cryptic plasmid	3,182												CP189687		

^*a*^ *Shigella* designations are the phylogroups as determined in Bengtsson et al. (13); *E. coli* pathotypes were determined in the study of Panchalingam et al. (4).

^*b*^ The shades of grey correspond to the different species isolates contained in each of the four subjects.

These data provide insight into the bacterial diversity of individual polymicrobial infections, as well as the specific organism genotype, which is important for understanding disease.

## Data Availability

All data have been released in BioProjects PRJNA1252136 and PRJNA1133736. The associated SRA and genome assembly accession numbers are listed in Table 1.
